# Supplementary material for: Testosterone exacerbates neutrophilia and cardiac injury in myocardial infarction via actions in bone marrow
Source: Nat Commun. 2025 Feb 5;16:1142. doi: 10.1038/s41467-025-56217-x (PMC11799197; doi:10.1038/s41467-025-56217-x)
Supplement: Supplementary file 1 — Supplementary Information [file 41467_2025_56217_MOESM1_ESM.pdf]

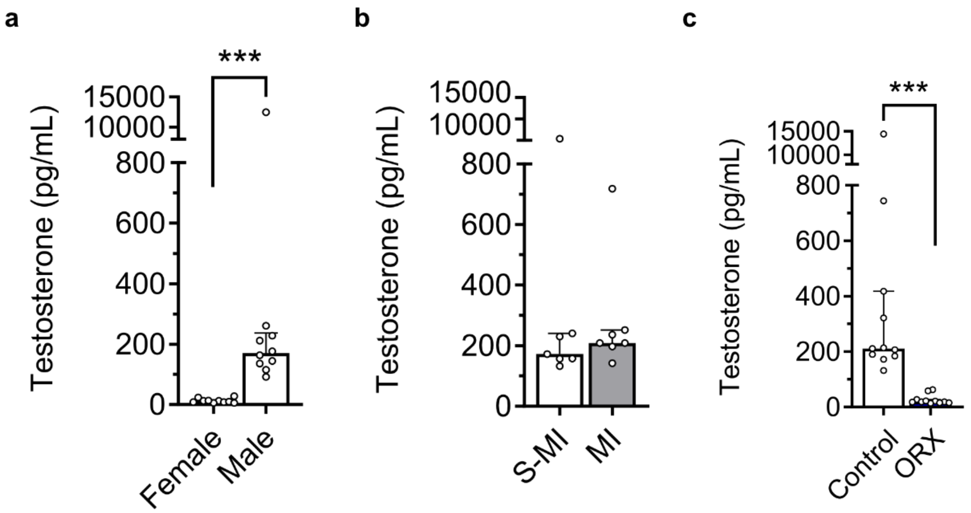

Supplementary Fig 1. Testosterone levels in mice. Testosterone concentrations were analyzed using high-sensitive gas chromatography-tandem mass spectrometry. **a**, Testosterone in plasma from female and male mice after 45 min ischemia followed by 24 h reperfusion (n = 10+10); **b**, in serum from mice after myocardial infarction (MI) induced by permanent ligation or sham-MI (S-MI) 48 h post-MI (n = 7+7) and **c,** in serum from castrated (ORX) or sham-castrated (control) male mice (n = 11+11). **a** and **c**, ***P <0.0001 (two-sided Mann Whitney). Bars indicate medians, error bars are interquartile ranges and circles represent individual mice. Source data are provided as Source data file.

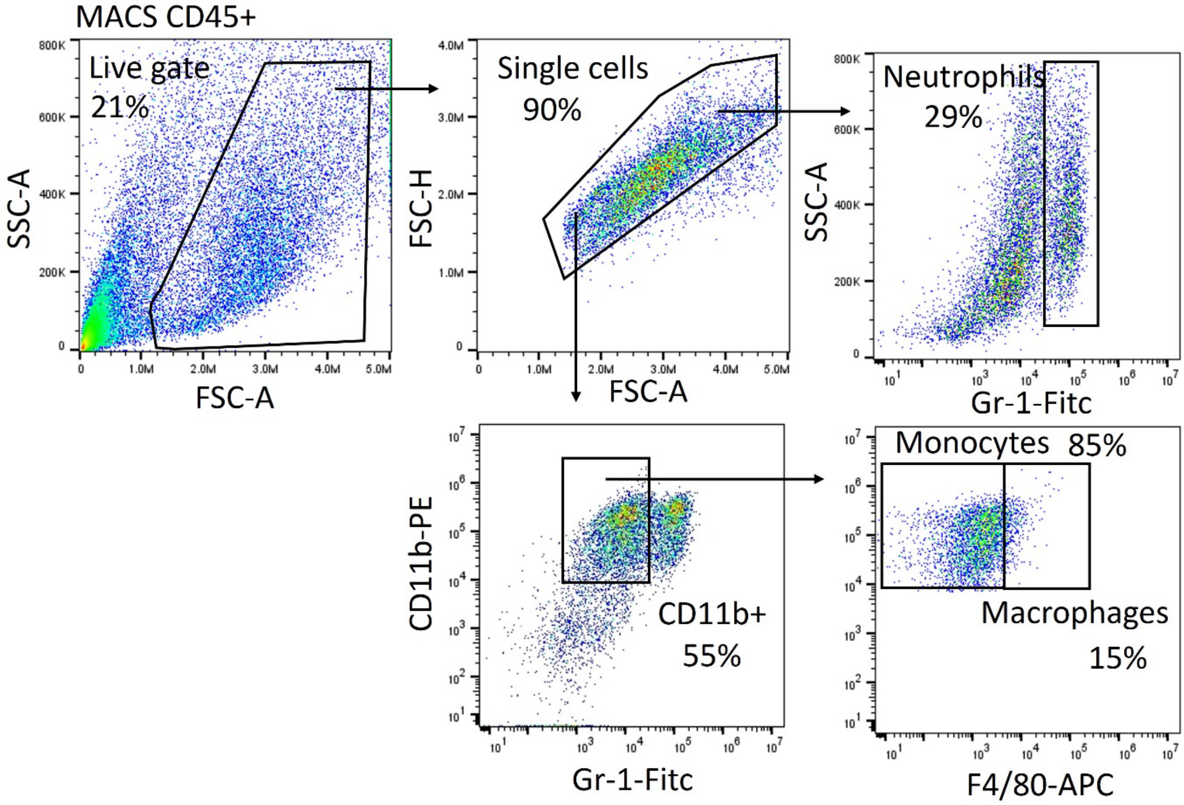

Supplementary Fig 2. Gating strategy for leukocytes in the heart. Gating strategy for neutrophils, monocytes and macrophages in heart tissue following CD45+ MACS enrichment.

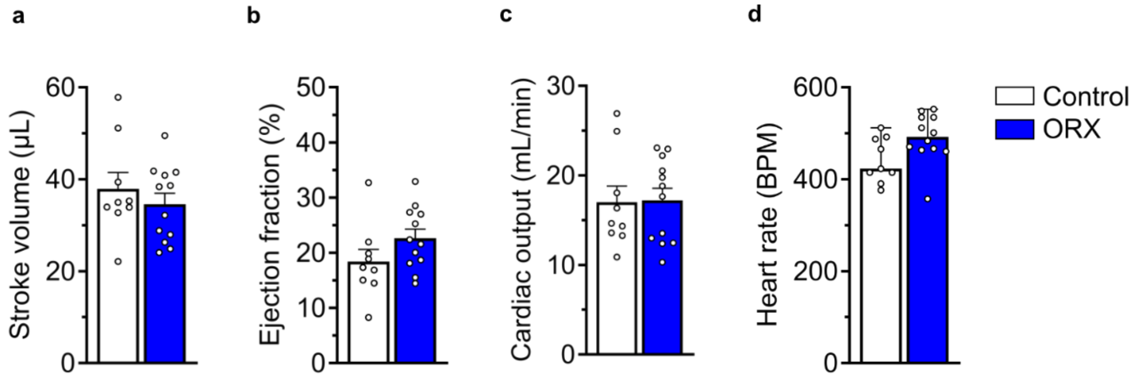

Supplementary Fig 3. Cardiac function in surviving castrated male mice 3 weeks post-myocardial infarction (MI). MI was induced by permanent ligation of the left coronary artery in castrated (ORX) or sham-castrated (control) mice. Echocardiography was performed 3 weeks post-MI and stroke volume **(a),** ejection fraction **(b),** **c**ardiac output **(c)** and heart rate **(d)** analyzed in surviving mice. BPM, beats/min; n = 9+12. Bars indicate means, error bars are SEM and circles represent individual mice. Source data are provided as Source data file.

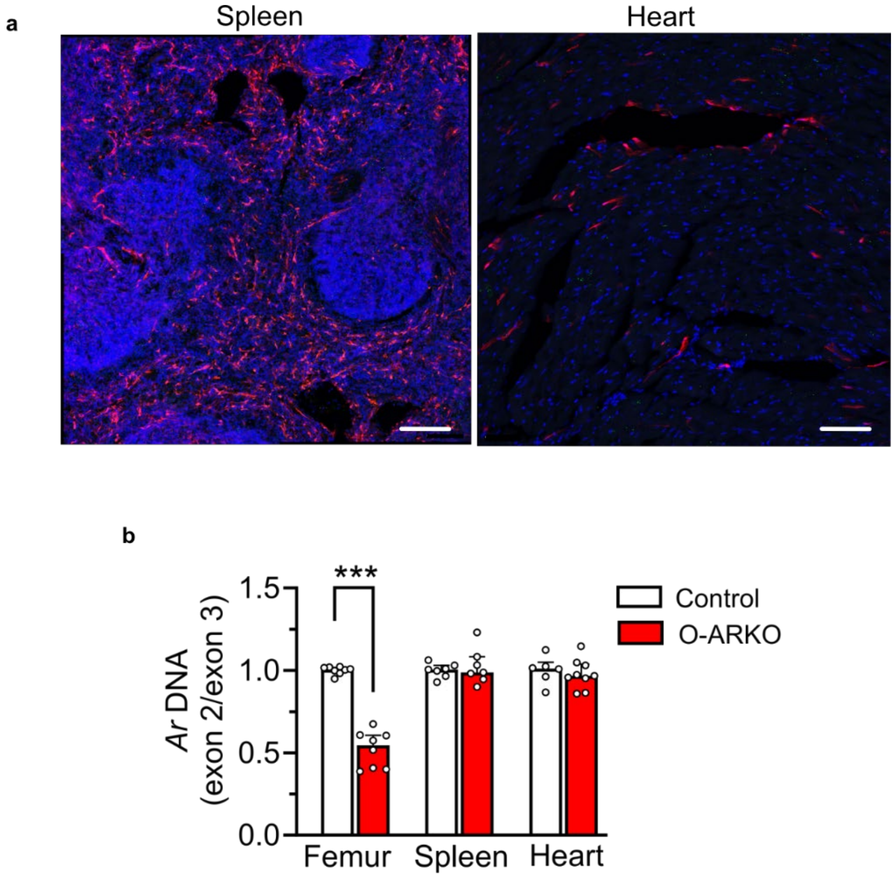

Supplementary Fig 4. Characterization of AR depletion in male mice with osterix-directed knockout of the androgen receptor (O-ARKO). a, Osterix-Cre-directed tdTomato reporter signal in spleen and heart of Osx1-Cre^{+} tdTomato fl/fl mice. tdTomato reporter (red) and Hoecht nuclear staining (blue). Scale bars spleen and heart 100 and 50 µm respectively. The staining was performed once. **b,** *Ar* DNA (ratio exon 2/exon 3) in femur shaft (n = 8+8), spleen (n = 7+7) and left heart ventricle (n = 6+9) in control and O-ARKO mice. ***P< 0.001 (two-sided Mann Whitney). Bars indicate medians, error bars are interquartile ranges and circles represent individual mice. Source data are provided as Source data file.

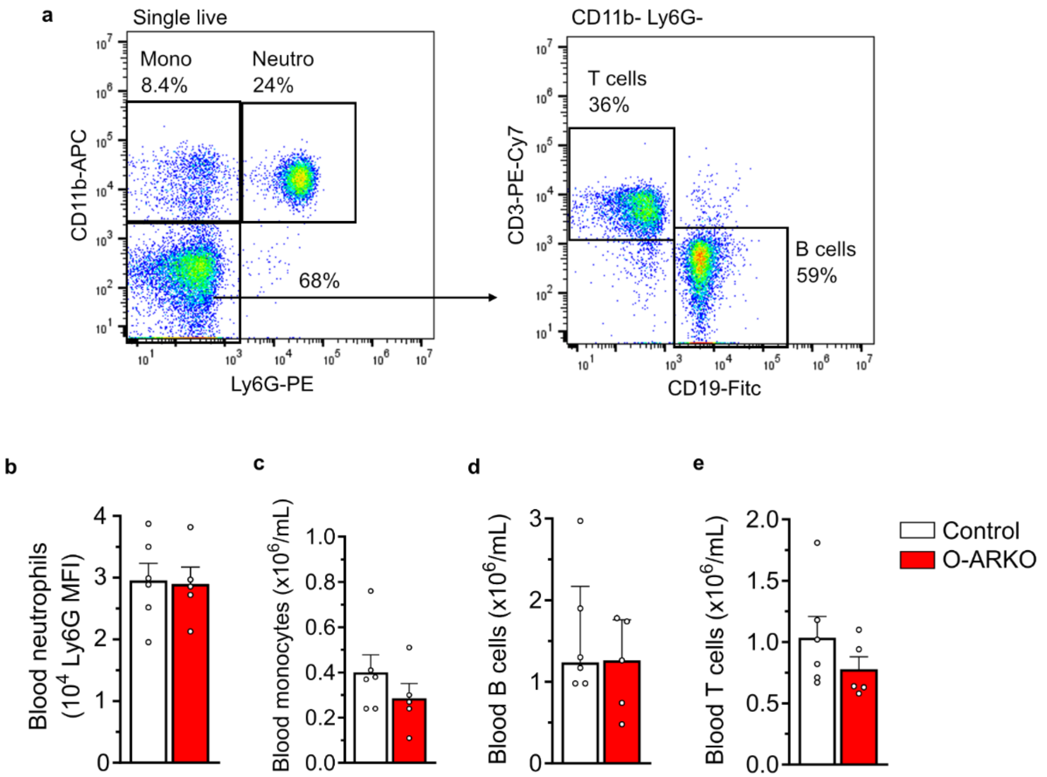

Supplementary Fig 5. Leukocyte counts in blood from osterix-directed knockout of the androgen receptor (O-ARKO) male mice after 45 min ischemia and 24 h reperfusion. a, Gating strategy for leukocytes in blood. Neutrophil Ly6G MFI (**b**), monocytes (**c**) B cells (**d**) and T cells (**e**) in blood from control and O-ARKO mice. **b-e**, n = 6+5. MFI, mean fluorescence intensity. Bars indicate means (**b-c**) or medians (**d-e**), error bars are SEM (**b-c**) or interquartile ranges (**d-e**) and circles represent individual mice. Source data are provided as Source data file.

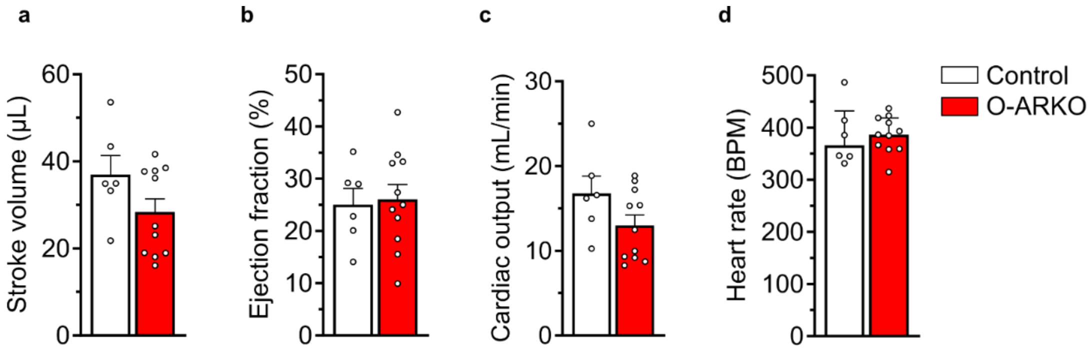

Supplementary Fig 6. Cardiac function in surviving O-ARKO (osterix-directed knockout of the androgen receptor) male mice, 3 weeks post-myocardial infarction (MI). a-d, MI was induced by permanent ligation of the left coronary artery in O-ARKO or control mice. Echocardiography was performed in surviving mice 3 weeks post-MI and stroke volume **(a)**, ejection fraction **(b)**, cardiac output **(c)** and heart rate **(d)** analyzed. BPM, beats/min. n = 6+11. Bars indicate means, error bars are SEM and circles represent individual mice. Source data are provided as Source data file.

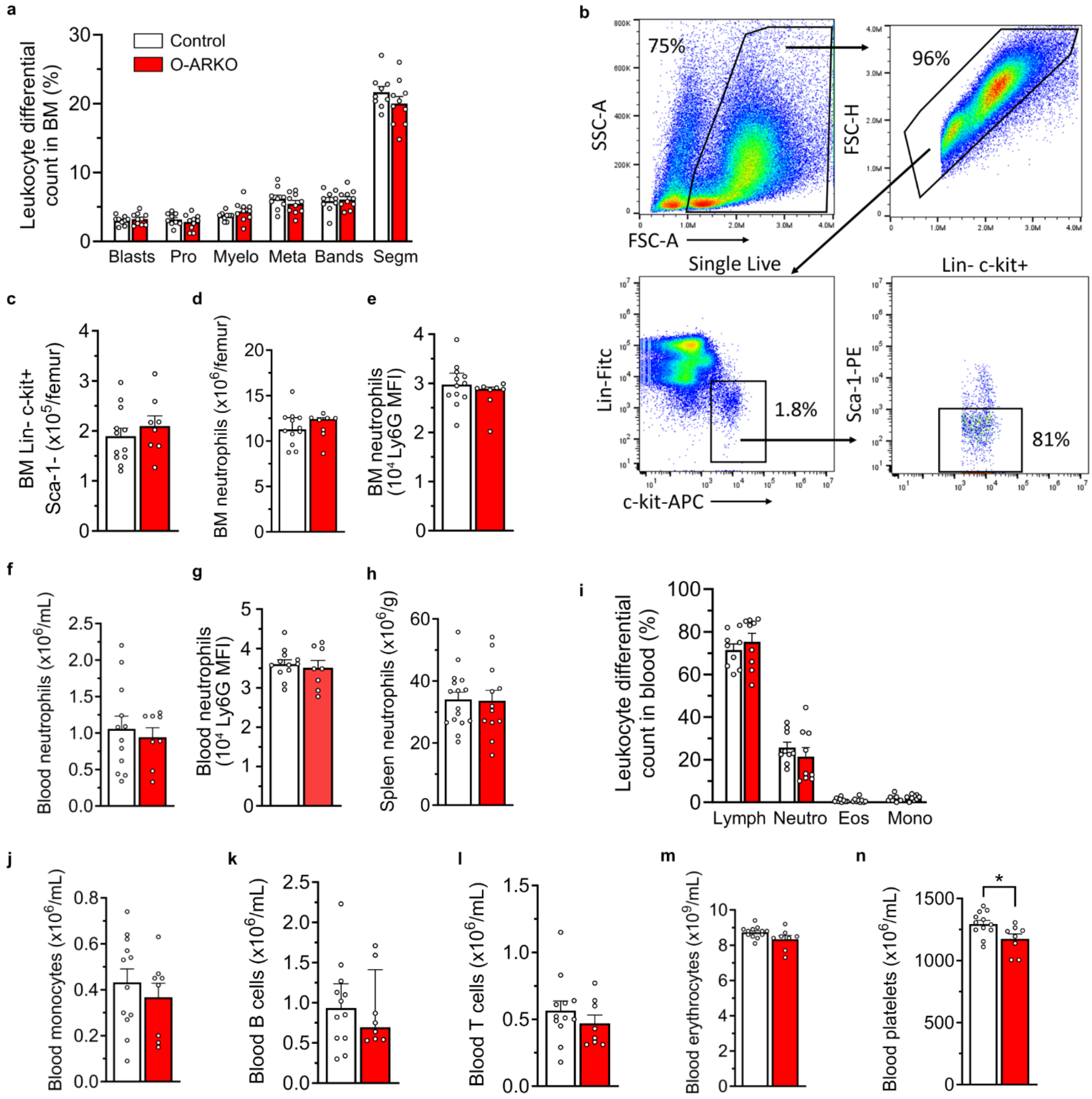

Supplementary Fig 7. Cell counts at steady-state in O-ARKO (osterix-directed knockout of the androgen receptor) male mice. a, Leukocyte differential count in BM analyzed by microscopy; promyelocytes (Pro), myelocytes (Myelo), metamyelocytes (Meta), band neutrophils (Bands), neutrophils with segmented nuclei (Segm); n = 9+10. **b,** Gating strategy for myeloid-committed hematopoietic stem and progenitor cells in bone marrow (BM). **c**, Common myeloid progenitors in BM gated as lineage negative (Lin-), c-kit+ and Sca-1-. **d-e**, Neutrophil total count and Ly6G MFI in BM analyzed by flow cytometry; **c-d**; n = 12+8. **f-h,** Neutrophil total count and Ly6G MFI in blood **(f-g)** and spleen **(h)** analyzed by flow cytometry; n = 12+8 in blood and 16+12 in spleen. **i,** Leukocyte differential count in blood analyzed by microscopy; lymphocytes (Lymph), neutrophils (Neutro), eosinophils (Eos), monocytes (Mono); n = 9+9. **j-n**, Total monocyte **(j),** B cell **(k)** and T cell counts in blood (**l**) analyzed by flow cytometry and erythrocyte **(m)** and platelet **(n)** counts in blood analyzed by Sysmex cell coounter. *P = 0.021 (two-sided unpaired Student t test); **j-n**; n = 12+8. MFI, mean fluorescence intensity. Bars indicate means (**a, c, f-j, l-n**) or medians (**d-e, k**), error bars are SEM (**a, c, f-j, l-n**) or interquartile ranges (**d-e, k**) and circles represent individual mice. Source data are provided as Source data file.

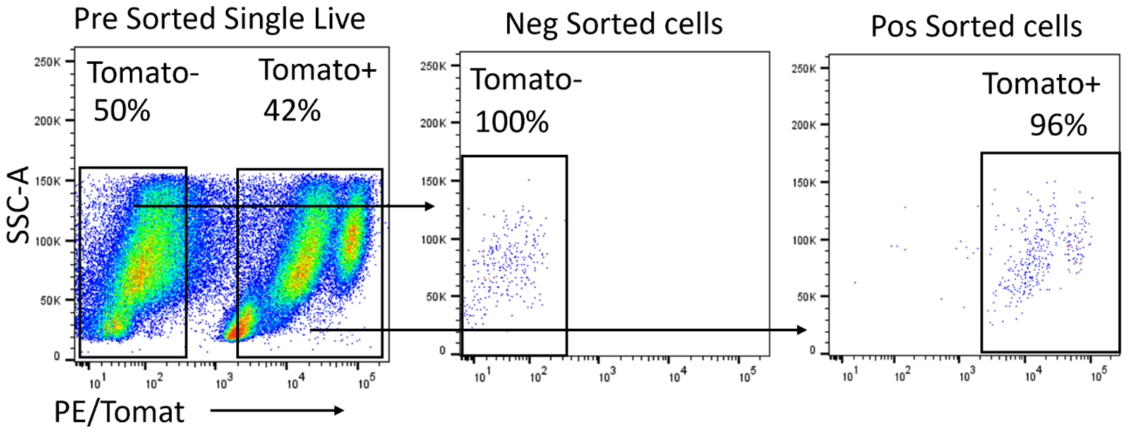

Supplementary Fig 8. Gating strategy for osx1-Cre^{+} tdTomato cell sorting.

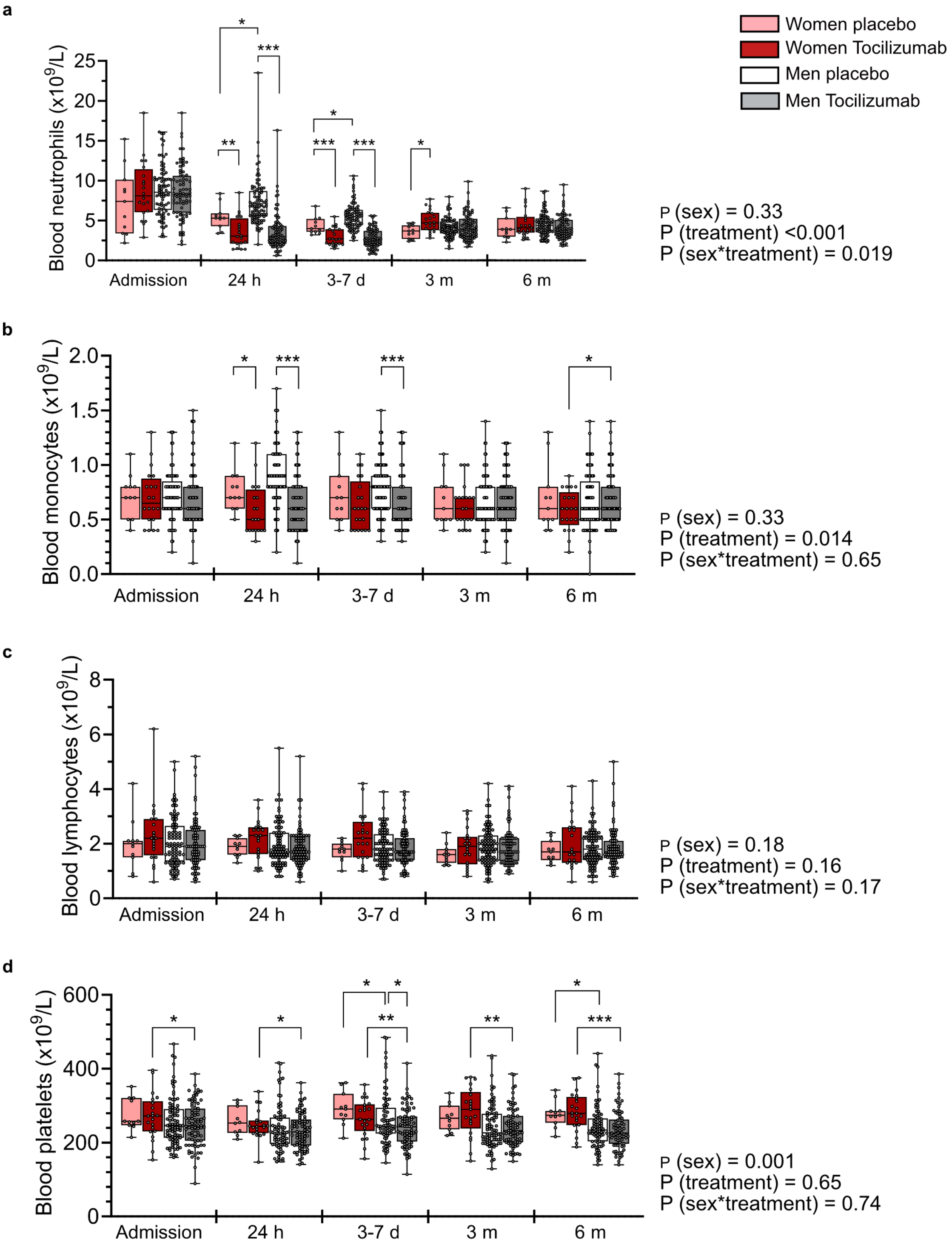

Supplementary Fig 9. Blood cell counts in the ASSAIL-MI study. Raw data from the double-blind ASSAIL-MI (ASSessing the effect of Anti-IL-6 treatment in MI) study. Patients (167 men, 32 women) with first-time ST elevation myocardial infarction (MI) were randomized to a single dose of the interleukin-6 receptor inhibitor tocilizumab or placebo prior to percutaneous coronary intervention. Men placebo; n = 87, men tocilizumab; n = 80, women placebo; n = 11 and women tocilizumab; n = 21. **a,** Neutrophils (raw data corresponding to estimated marginal means in Fig 4b). **b,** monocytes**, c,** lymphocytes and **d**, platelets. Box boundaries represent 25th to 75th percentile, horizontal bars represent median values, whiskers represent minimum and maximum values. Dots represent individual participant values. P-values are from age-adjusted two-sided repeated measures ANOVA with LSD post hoc tests. *P<0.05, **P<0.01, ***P<0.001.

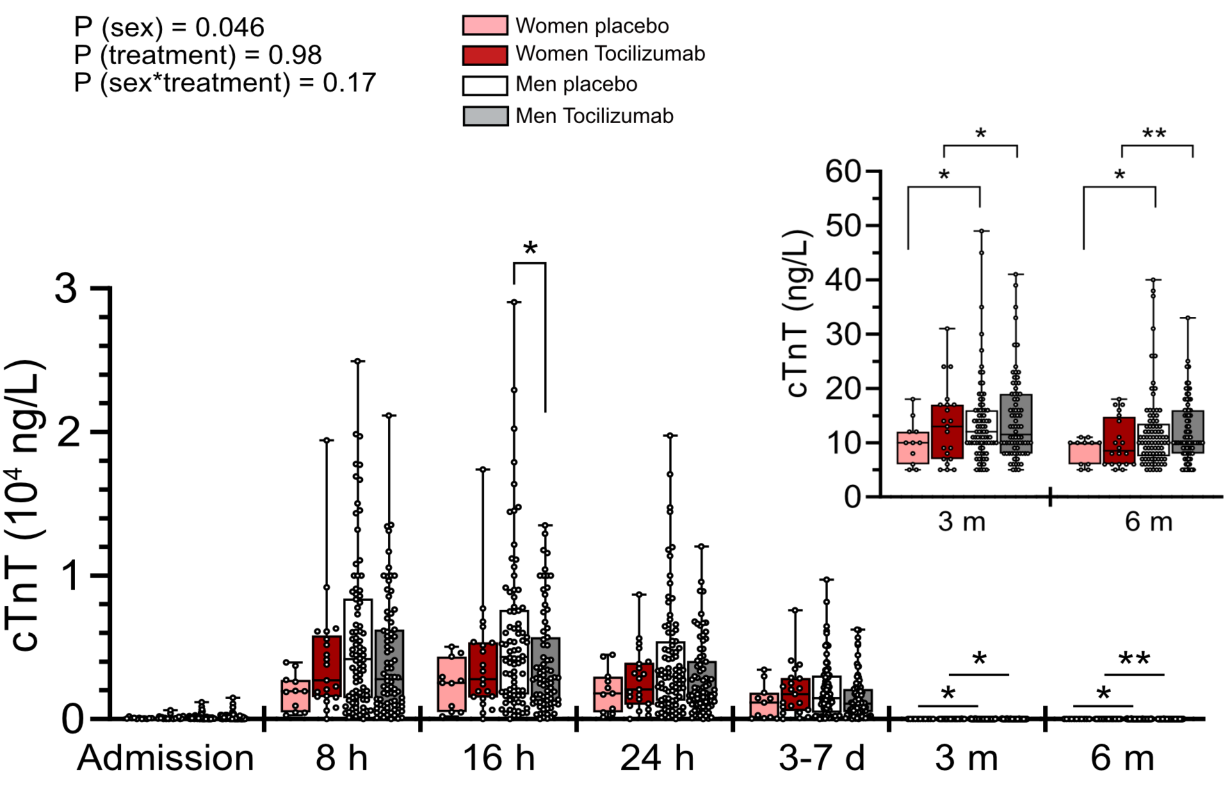

Supplementary Fig 10. Cardiac troponin T (cTnT) levels in the ASSAIL-MI study. Raw data from the double-blind ASSAIL-MI (ASSessing the effect of Anti-IL-6 treatment in MI) study. Patients (167 men, 32 women) with first-time ST elevation myocardial infarction (MI) were randomized to a single dose of the interleukin-6 receptor inhibitor tocilizumab or placebo prior to percutaneous coronary intervention. Men placebo; n = 87, men tocilizumab; n = 80, women placebo; n = 11 and women tocilizumab; n = 21**.** Troponin T was analyzed at admission and 8 h, 16 h, 24 h, 3-7 days, 3 months and 6 months after reperfusion. Box boundaries represent 25th to 75th percentile, horizontal bars represent median values, whiskers represent minimum and maximum values. Dots represent individual participant values. P-values are from age-adjusted two-sided repeated measures ANOVA with LSD post hoc tests. *P<0.05, **P<0.01.

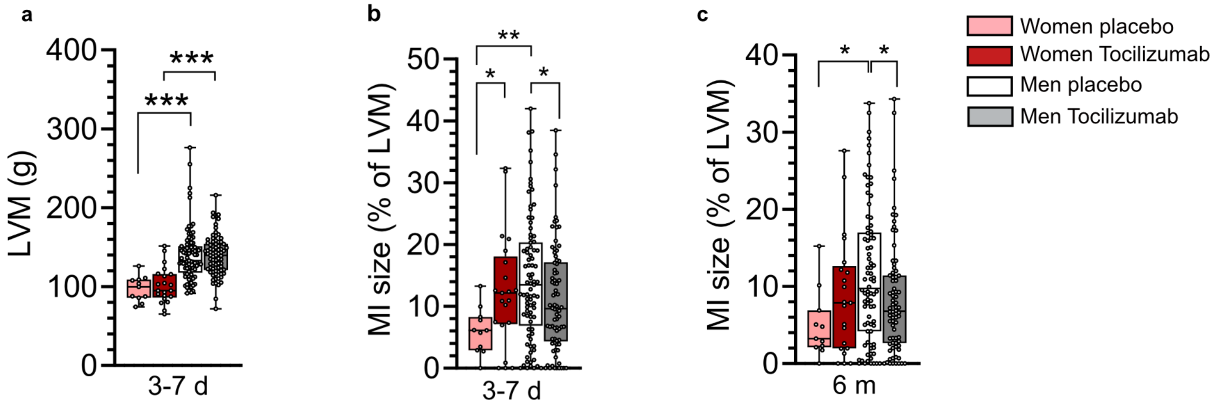

P (sex) <0.001
P (treatment) = 0.53
P (sex*treatment) = 0.71

P (sex) = 0.029
P (treatment) = 0.50
P (sex*treatment) = 0.012

P (sex) = 0.034
P (treatment) = 0.96
P (sex*treatment) = 0.038

Supplementary Fig 11. Cardiac magnetic resonance imaging data in the ASSAIL-MI study. Raw data from the double-blind ASSAIL-MI (ASSessing the effect of Anti-IL-6 treatment in MI) study. Patients (167 men, 32 women) with first-time ST elevation myocardial infarction (MI) were randomized to a single dose of the interleukin-6 receptor inhibitor tocilizumab or placebo prior to percutaneous coronary intervention. Men placebo; n = 87, men tocilizumab; n = 80, women placebo; n = 11 and women tocilizumab; n = 21. **a**, Left ventricular mass (LVM; raw data corresponding to estimated marginal means in Fig 4c), **b**, MI size at 3-7 d (corresponding to Fig 4d) and **c**, MI size at 6 m (corresponding to Fig 4e). Box boundaries represent 25th to 75th percentile, horizontal bars represent median values, whiskers represent minimum and maximum values. Dots represent individual participant values. P-values are from age-adjusted two-sided repeated measures ANCOVA with LSD post hoc tests. *P<0.05, ***P<0.001.

Supplementary Table 1. Baseline characteristics in the ASSAIL-MI trial stratified by sex and treatment allocation

	Men Placebo (n=87) **A**	Men Tocilizumab (n=80) **B**	Women Placebo (n=11) **C**	Women Tocilizumab (n=21) **D**	*P*
Demographics					
Age, yrs	59 ± 9	61 ± 10	62 ± 5	66 ± 9	A-D: 0.032
Body mass index, kg/m^2	27.9 ± 4.0	27.4 ± 4.3	24.6 ± 4.9	25.9 ± 5.2	NS
White	83 (95)	79 (99)	11 (100)	20 (95)	NS
Smoking status					
Never smokers	33 (38)	29 (36)	3 (27)	9 (43)	NS
Previous smokers	22 (25)	26 (33)	2 (18)	7 (33)	NS
Current smokers	32 (37)	25 (31)	6 (55)	5 (24)	NS
Prior conditions					
Angina pectoris	0 (0)	1 (1)	1 (9)	0 (0)	A-C: 0.005
Cerebrovascular disease	1 (1)	3 (4)	1 (9)	1 (5)	NS
Other vascular disease	3 (3)	0 (0)	0 (0)	1 (5)	NS
Diabetes mellitus	6 (7)	6 (8)	0 (0)	2 (10)	NS
Hypertension	24 (28)	24 (30)	6 (55)	9 (43)	NS
Treatment					
ACE inhibitor or ARB	22 (25)	18 (23)	3 (27)	4 (19)	NS
Aldosterone antagonist	1 (1)	0 (0)	0 (0)	0 (0)	NS
Oral anticoagulants	2 (2)	5 (6)	0 (0)	0 (0)	NS
Platelet inhibitor	4 (5)	8 (10)	1 (9)	4 (19)	NS
Beta-blocker	2 (2)	5 (6)	1 (9)	3 (14)	NS
Calcium antagonist	8 (9)	9 (11)	2 (18)	4 (19)	NS
Diuretic	7 (8)	5 (6)	1 (9)	3 (14)	NS
Statin	7 (8)	14 (18)	2 (18)	5 (24)	NS
Up-front DAPT	87 (100)	80 (100)	11 (100)	21 (100)	NS
Clinical characteristics					
Blood pressure at admission, mm Hg					
Systolic	133 ± 22	131 ± 22	126 ± 19	132 ± 28	NS
Diastolic	86 ± 16	82 ± 14	74 ± 15	79 ± 24	NS
Heart rate at admission, beats/min	73 ± 18	72 ± 16	73 ± 15	66 ± 12	NS
Time from symptom onset to arrival at PCI center, min	147 ± 72	155 ± 80	168 ± 72	136 ±55	NS
Door-to-balloon time, min	22 ± 10	23 ± 10	30 ± 13	22 ± 6	NS
Killip class					
I	85 (98)	76 (95)	10 (91)	20 (95)	NS
II	2 (2)	3 (4)	1 (9)	1 (5)	NS
III	0 (0)	0 (0)	0 (0)	0 (0)	NS
IV	0 (0)	1(1)	0 (0)	0 (0)	NS
GRACE risk score	135 ± 21	139 ± 25	141 ± 21	144 ± 25	NS
Infarct location					
Left anterior descending branch	35 (40)	32 (40)	1 (9)	6 (29)	NS
Circumflex or marginal	10 (11)	9 (11)	3 (27)	2 (10)	NS
Right coronary artery	39 (45)	35 (44)	7 (64)	12 (57)	NS
Other	3 (3)	4 (5)	0 (0)	1 (5)	NS
Laboratory values					
Hemoglobin, g/l	146 ± 11	147 ±12	134 ± 18	131 ± 10	A-C: 0.015<br>B-D: <0.001
Platelet count, 10^9/l	258 ± 64	249 ±58	276 ± 43	270 ±62	NS
Total white blood cell count, 10^9/l	11.7 ±3.3	11.5 ± 3.3	10.5 ± 3.8	11.8 ± 4.1	NS
Aspartate transaminase, U/l	31 (24-38)	28 (22-43)	27 (22-34)	29 (22-33)	NS
Troponin T, ng/l	49 (31-105)	43 (22-174)	58 (25-76)	53 (32-147)	NS
CK-MB, μg/l	5.3 (3.0-11.0)	5.0 (2.6-16.0)	6.0 (2.2-7.1)	5.0 (3.0-6.6)	NS
NT-proBNP, ng/l	63 (50-109)	61 (50-157)	177 (58-347)	198 (77-436)	B-D: 0.007
Creatinine, mmol/l	81 ± 20	76 ±17	58 ± 12	63 ± 12	A-C:<0.001<br>B-D: 0.017
Glucose, mmol/l	8.8 ± 2.9	8.6 ± 2.4	8.1 ± 2.5	9.3 ± 4.8	NS
HbA1c, mmol/mol	37 (34-40)	37 (34-41)	37 (33-38)	38 (33-40)	NS
Total cholesterol, mmol/l	5.1 ± 0.9	5.2 ± 1.2	5.9 ± 1.2	5.4 ± 1.3	NS
HDL cholesterol, mmol/l	1.1 (0.9-1.3)	1.1 (0.9-1.3)	1.4 (1.3-1.8)	1.2 (1.1-1.6)	A-C: 0.008
LDL cholesterol, mmol/l	3.7 ± 0.8	3.6 ± 1.0	4.1 ± 1.0	3.9 ± 1.2	NS
C-reactive protein, mg/l	2.6 (1.4-5.0)	2.2 (0.9-5.0)	4.1 (2.9-5.0)	3.1 (1.0-7.4)	NS
Albumin, g/l	42 ± 3	42 ± 3	43 ± 7	40 ± 4	NS

Baseline characteristics (at admission) in the ASSAIL-MI (Assessing the effect of Anti-IL-6 treatment in MI) study. Values are mean ± SD, n (%), or median (interquartile range). Depending on sample distribution, data were analyzed using one-way ANOVA with Bonferroni as post hoc test or Kruskal-Wallis with Mann-Whitney as post hoc test. Categorical variables were tested using Chi-square. P-values are from post hoc testing. P > 0.05 are denoted as not significant (NS). ACE = angiotensin-converting enzyme; ARB = angiotensin receptor blocker; CK-MB = creatine kinase myocardial band; DAPT = dual anti-platelet therapy; HDL = high-density lipoprotein; LDL = low-density lipoprotein; PCI = percutaneous coronary intervention; NT-proBNP = N-terminal pro-B-type natriuretic peptide.

Supplementary Table 2. Antibody list

Antibody	Clone	Supplier	Cat no.	Lot. no.	RRID	Conc of antibody for 10^6 cells stained in 200 µL (µg/mL)
Rat anti-mouse CD16/CD32	2.4G2	BD Pharmingen	553142	0028326	AB_394656	5.0
Rat anti-mouse CD45-AF488	30-F11	Biolegend	103122	B343093	AB_493531	2.5
Rat anti-mouse CD11b-PE-Cy7	M1/70	Biolegend	101216	B316177	AB_312799	1.0
Rat anti-mouse CD11b-APC	M1/70	Biolegend	101212	B352669	AB_312795	1.0
Rat anti-mouse CD11b-PE	M1/70	Biolegend	101207	B155006	AB_312790	2.0
Rat anti-mouse Ly6G-AF488	1A8	Biolgend	127626	B329584	AB_2561340	5.0
Rat anti-mouse Ly6G-PE-Cy7	1A8	Biolegend	127618	B351626	AB_1877261	2.0
Rat anti-mouse Ly6G-PE	1A8	Biolegend	127608	B235377	AB_1186099	1.0
Rat anti mouse CD3-PE-Cy7	17A2	Biolegend	100220	B284568	AB_1732057	2.0
Rat anti CD19-Fitc	1D3	BD Biosciences	553785	3204623	AB_396681	5.0
Rat anti c-kit-APC	2B8	eBioscience	17-1171	E029572	AB_469431	1.0
Rat anti Sca-1-PE	D7	eBioscience	12-5981	E026399	AB_466085	1.0
Rat anti-mouse Gr-1-Fitc	RB6-8C5	eBioscience	11-5931	E00739-1632	AB_465314	5.0
Rat anti-mouse F4/80-APC	BM8	eBioscience	17-4801	E212349	AB_2784648	2.0
Rat anti-mouse CD45 MACS beads	30-F11.1	Miltenyi	130-052-301	5221207062	AB_2877061	10 µL/10^7 cells
Rat anti mouse TER119 MACS beads	TER-119	Miltenyi	130-049-901	5220604307	AB_2936424	10 µL/10^7 cells
Fitc labelled anti mouse Lineage cocktail with isotype control		Biolegend	133302	B341885		20 µL/10^6 cells
