# Supplementary material for: Testosterone exacerbates neutrophilia and cardiac injury in myocardial infarction via actions in bone marrow
Source: Nat Commun. 2025 Feb 5;16:1142. doi: 10.1038/s41467-025-56217-x (PMC11799197; doi:10.1038/s41467-025-56217-x)
Supplement: Supplementary file 2 — Reporting Summary [file 41467_2025_56217_MOESM2_ESM.pdf]

nature portfolio

Corresponding author(s): Åsa Tivesten

Last updated by author(s): Jan 10, 2025

Reporting Summary

Nature Portfolio wishes to improve the reproducibility of the work that we publish. This form provides structure for consistency and transparency in reporting. For further information on Nature Portfolio policies, see our Editorial Policies and the Editorial Policy Checklist.

Statistics

For all statistical analyses, confirm that the following items are present in the figure legend, table legend, main text, or Methods section.

n/a	Confirmed	
☐	☒	The exact sample size (*n*) for each experimental group/condition, given as a discrete number and unit of measurement
☐	☒	A statement on whether measurements were taken from distinct samples or whether the same sample was measured repeatedly
☐	☒	The statistical test(s) used AND whether they are one- or two-sided<br>*Only common tests should be described solely by name; describe more complex techniques in the Methods section.*
☐	☒	A description of all covariates tested
☐	☒	A description of any assumptions or corrections, such as tests of normality and adjustment for multiple comparisons
☐	☒	A full description of the statistical parameters including central tendency (e.g. means) or other basic estimates (e.g. regression coefficient) AND variation (e.g. standard deviation) or associated estimates of uncertainty (e.g. confidence intervals)
☐	☒	For null hypothesis testing, the test statistic (e.g. *F*, *t*, *r*) with confidence intervals, effect sizes, degrees of freedom and *P* value noted<br>*Give P values as exact values whenever suitable.*
☒	☐	For Bayesian analysis, information on the choice of priors and Markov chain Monte Carlo settings
☒	☐	For hierarchical and complex designs, identification of the appropriate level for tests and full reporting of outcomes
☒	☐	Estimates of effect sizes (e.g. Cohen's *d*, Pearson's *r*), indicating how they were calculated

Our web collection on statistics for biologists contains articles on many of the points above.

Software and code

Policy information about availability of computer code

Data collection	Echocardiography in mice: VEVO2100, VEVO3100, or VEVO770 echocardiography system. Cardiac magnetic resonance imaging in human: 1.5-T systems, Siemens Avanto, Philips Ingenia. Human blood analysis: MODULAR platform, Roche Diagnostics and Elecsys 2010 analyzer, Roche Diagnostics. Mouse plasma analysis: Spectramax i3 and SoftMaxPro vers 7.1 or an Alinity, Abbott Laboratories. Flowcytometry: Accuri C6 and CFlow vers 1.0.227.4, FACS Aria and BD FACSDiva vers 8.0 or FACS Aria Fusion and BD FACSDiva 9.0. Microscopy: Axiophot and AxioVision vers 4.8.2 or Leica SP8 Laser confocal and Nikon Spinning Disk confocal microscope. qPCR: Viia7 and QuantStudio Real-Time PCR System vers 1.3. Single cell RNA seq analysis: Seurat/4.1.010 within R/4.1.3 (R Core Team, 2022).
Data analysis	Echocardiography in mice: Vevo® LAB desktop software version 5.5.0. Cardiac magnetic resonance imaging in human: Segment software, Medviso, Lund, Sweden. Flowcytometry: FlowJo vers 10.9. Statistical analysis: GraphPad Prism vers 10.2.3. The software "R": A language and environment for statistical computing, R Foundation for Statistical Computing, Vienna, Austria.<br><br>The R code used for analysis of mouse single cell gene expression data has been deposited to a GitHub repository (DOI:10.5281/zenodo.14221831).

For manuscripts utilizing custom algorithms or software that are central to the research but not yet described in published literature, software must be made available to editors and reviewers. We strongly encourage code deposition in a community repository (e.g. GitHub). See the Nature Portfolio guidelines for submitting code & software for further information.

Data

Policy information about availability of data

All manuscripts must include a data availability statement. This statement should provide the following information, where applicable:

- Accession codes, unique identifiers, or web links for publicly available datasets
- A description of any restrictions on data availability
- For clinical datasets or third party data, please ensure that the statement adheres to our policy

Source data are provided with this paper. All mouse data generated or analysed during this study are included in this published article (and its supplementary information files).

Ethical restrictions from the Regional Committee for Medical and Research Ethics in Southeast Norway prohibit data from individual patients from being made available on publicly available repositories. However, an institutional data transfer agreement can be established, and data can be shared if the aims of data use are covered by ethical approval and patient consent. The procedure will involve an update to the ethical approval as well as a review by legal departments at both institutions, and the process will typically take 2 to 4 months from initial contact. Access requests may be sent to Kaspar Broch (sbbrok@ous-hf.no).

The annotated mouse single cell gene expression data to evaluate Ar and Cxcl12 expression in stromal cells were retrieved from GEO (GSE128423; https://www.ncbi.nlm.nih.gov/geo/query/acc.cgi?acc=GSE128423).

Research involving human participants, their data, or biological material

Policy information about studies with human participants or human data. See also policy information about sex, gender (identity/presentation), and sexual orientation and race, ethnicity and racism.

Reporting on sex and gender	Assigned sex was specified in the description of all data from human participants.
Reporting on race, ethnicity, or other socially relevant groupings	Race (white/non-white) of human participants is provided in Supplementary Table 1.
Population characteristics	Population were patients admitted to three involved PCI centers in Norway with first-time ST-Elevated myocardial infarction (STEMI) with symptom onset less than 6 hours before PCI. Demographic details of participants are provided in Supplementary Table 1.
Recruitment	Patients were screened for eligibility and recruitment was done upon admittance due to acute STEMI at either participating site. There was no participant compensation.
Ethics oversight	The trial protocol was approved by the regional ethics committee (Regional Committee for Medical Research Ethics South East Norway; 2016/1223-1), and all participants provided written informed consent. An independent Data and Safety Monitoring Board oversaw the safety of the trial. The trial was conducted in compliance with the declaration of Helsinki and with the rules outlined in the guidelines for Good Clinical Practice.

Note that full information on the approval of the study protocol must also be provided in the manuscript.

Field-specific reporting

Please select the one below that is the best fit for your research. If you are not sure, read the appropriate sections before making your selection.

☒ Life sciences ☐ Behavioural & social sciences ☐ Ecological, evolutionary & environmental sciences

For a reference copy of the document with all sections, see nature.com/documents/nr-reporting-summary-flat.pdf

Life sciences study design

All studies must disclose on these points even when the disclosure is negative.

Sample size	No method was used to pre-determine sample sizes; we designed our experiments based on previous experience. All sample sizes are given in the respective figure legends and/or methods.
Data exclusions	No data points were excluded.
Replication	All experiments were performed using at least four biological replicates and specific sample sizes are mentioned in the figure legends. Most experiments contain statistical analysis and results of statistical testing are indicated in figure legends.
Randomization	All mice and humans were randomly allocated into experimental groups.
Blinding	All data analysis was performed by a blinded investigator.

Reporting for specific materials, systems and methods

We require information from authors about some types of materials, experimental systems and methods used in many studies. Here, indicate whether each material, system or method listed is relevant to your study. If you are not sure if a list item applies to your research, read the appropriate section before selecting a response.

Materials & experimental systems

n/a	Involved in the study
☐	☒ Antibodies
☒	☐ Eukaryotic cell lines
☒	☐ Palaeontology and archaeology
☐	☒ Animals and other organisms
☐	☒ Clinical data
☒	☐ Dual use research of concern
☒	☐ Plants

Methods

n/a	Involved in the study
☒	☐ ChIP-seq
☐	☒ Flow cytometry
☒	☐ MRI-based neuroimaging

Antibodies

Antibodies used

Rat anti-mouse CD16/CD32 (clone 2.4G2), BD Pharmingen, cat.no. 553142, RRID: AB_394656, dil 1:100
Rat anti-mouse CD45-AF488 (clone 30-F11), Biolegend, cat.no. 103122, RRID: AB_493531, dil 1:200
Rat anti-mouse CD11b-PE-Cy7 (clone M1/70, Biolegend, cat.no. 101216, RRID: AB_312799, dil 1:200
Rat anti-mouse CD11b-APC (clone M1/70), Biolegend, cat.no. 101212, RRID: AB_312795, dil 1:200
Rat anti-mouse CD11b-PE (clone M1/70), Biolegend, cat.no. 101207, RRID: AB_312790, dil1:100
Rat anti-mouse Ly6G-AF488 (clone 1A8), Biolgend, cat.no. 127626, RRID: AB_2561340, dil 1:100
Rat anti-mouse Ly6G-PE-Cy7 (clone 1A8), Biolegend, cat.no. 127618, RRID: AB_1877261, dil 1:100
Rat anti-mouse Ly6G-PE (clone 1A8), Biolegend, cat.no. 127608, RRID: AB_1186099, dil 1:200
Rat anti mouse CD3-PE-Cy7 (clone 17A2), Biolegend, cat.no.100220, RRID: AB_1732057, dil 1:100
Rat anti CD19-Fitc (clone 1D3), BD Biosciences, cat.no. 553785, RRID: AB_396681, dil 1:100
Rat anti c-kit-APC (clone 2B8), eBiosciences, cat.no. 17-1171, RRID: AB_469431, dil 1:200
Rat anti Sca-1-PE (clone D7, eBiosceneces, cat.no. 12-5981, RRID: AB_466085, dil 1:200
Fitc labelled anti mouse Lineage cockatil, Biolegend, cat.no. 133302, 20 uL/10^6 cells
Rat anti-mouse Gr-1-Fitc (clone RB6-8C5), eBioscience, cat.no. 11-5931, RRID: AB_465314, dil 1:100
Rat anti-mouse F4/80-APC (clone BM8), eBioscience, cat.no. 17-4801, RRID: AB_2784648, dil 1:100
Rat anti-mouse CD45 MACS beads (clone 30F11.1), Miltenyi, cat.no. 130-052-301, RRID: AB_2877061, 10 uL/10^7 cells
Rat anti mouse TER119 MACS beads (clone TER-119), Miltenyi, cat.no. 130-049-901, RRID: AB_2936424, 10 uL/10^7 cells

Validation

All antibodies were validated by the supplier.

Animals and other research organisms

Policy information about studies involving animals; ARRIVE guidelines recommended for reporting animal research, and Sex and Gender in Research

Laboratory animals

All mice used in the project were on C57BL/6J background (Jackson Laboratory, Ben Harbor, ME, USA; JAX000664). Male mice with Osterix (Osx1)-Cre-mediated inactivation of the androgen receptor (O-ARKO mice) were generated by breeding androgen receptor (AR)+/flox female mice1 with male Osx1-Cre+ mice (Jackson laboratory, Bar Harbor, Maine, USA; JAX006361). Male tdTomato+ Osx1-Cre+ mice were generated by breeding homozygous tdTomato+/+ mice (Rosa26-CAG-loxP-stop-loxP-tdTomato; Jackson laboratory, Bar Harbor, Maine, USA; JAX007914) with Osx1-Cre+ mice.
Considering our scientific focus we mainly used male mice. Female mice were used in one experiment. The age of the mice in the project were 12-20 weeks, but the ages within one experiment were always matched.
The mice were housed in a temperature- and humidity–controlled room with a 7:00-19:00 h light cycle and consumed a soy-free diet (RM3(E)-soy free, SDS; Teklad Global 16% Protein Rodent diet 2016, Harlan Laboratories or R70, Lantmännen) and tap water ad libitum.

Wild animals

This project did not involve wild animals.

Reporting on sex

Sex is specified in all experiments in this study.

Field-collected samples

This project did not involve field-collected samples.

Ethics oversight

The Ethics Committee on Animal Care and Use in Gothenburg had approved all procedures. Ethical approval numbers are 8/2014 and 3007/2020.

Note that full information on the approval of the study protocol must also be provided in the manuscript.

Clinical data

Policy information about clinical studies

All manuscripts should comply with the ICMJE guidelines for publication of clinical research and a completed CONSORT checklist must be included with all submissions.

Clinical trial registration	Clinicaltrials.gov, identifier NCT03004703
Study protocol	The major description of the study protocol is described in Anstensrud AK, Woxholt S, Sharma K, et al. Rationale for the ASSAIL-MI-trial: a randomised controlled trial designed to assess the effect of tocilizumab on myocardial salvage in patients with acute ST-elevation myocardial infarction (STEMI). Open Heart 2019;6:e001108. doi: 10.1136/openhrt-2019-001108
Data collection	The study was preformed during March 16th 2017 and February 13th 2020 data on clinical variables, blood samples and magnetic resonance imaging (MRI) were collected in this period.
Outcomes	The primary endpoint of the clinical trial was myocardial salvage index based on MRI. Secondary endpoints used in this sub-study was based on MRI. Neutrophil count was an exploratory endpoint.

Plants

Seed stocks	Not applicable
Novel plant genotypes	Not applicable
Authentication	Not applicable

Flow Cytometry

Plots

Confirm that:

☒ The axis labels state the marker and fluorochrome used (e.g. CD4-FITC).

☒ The axis scales are clearly visible. Include numbers along axes only for bottom left plot of group (a 'group' is an analysis of identical markers).

☒ All plots are contour plots with outliers or pseudocolor plots.

☒ A numerical value for number of cells or percentage (with statistics) is provided.

Methodology

Sample preparation	Blood for flow cytometry was drawn in K3-EDTA Micro tubes (Sarstedt). To obtain single bonemarrow and spleen cells for flow cytometry, tissue was passed through a 70 µm cell strainer. To remove erythrocytes from bonemarrow, spleen and blood, cells were lyzed for 5 min in ammonium chloride buffer (0.16 M NH4Cl, 0.13 M EDTA and 12 mM NaHCO3 in H2O), washed and resuspended in FACS buffer (2% heat-inactivated FCS and 2 mM EDTA in PBS), passed through a 70 µm cell strainer and counted in a Sysmex KX-21 (Sysmex Corporation).<br><br>CD45+ leukocytes from heart were isolated from left ventricle and septum. Tissue was minced in a small volume of digestion buffer: collagenase I, 450 U/mL (Sigma); collagenase XI, 125 U/mL (Sigma); hyaluronidase I-S, 60 U/mL (Sigma) and DNase, 60 U/mL (Worthington) and digested in 1 mL buffer with mixing (1400 rpm) for 1 h at 37ºC. The digested tissue was passed through a 70 µm cell strainer using a 5 mL syringe plunger. Cells were collected and washed in flow cytometry (FACS) buffer (2% FBS and 2 mM EDTA in PBS without Mg2+/Ca2+), stained and sorted according to the manufacturer´s instructions using anti-mouse CD45 conjugated MACS® MicroBeads (Miltenyi Biotec, #130-052-301, clone 30F11.1) and LS columns (Miltenyi Biotec, #130-042-401). The cells in the CD45 positive fraction were counted with a NucleoCounter NC-100 (Chemimetec). Immuno staining for flow cytometry: After Fc-blockage with anti-mouse CD16/CD32 (clone 2.4G2, BD Bioscience 553142), expression of various cell-surface markers was detected using various fluorochrome-conjugated antibodies.
Instrument	Accuri C6, FACS Aria or FACS Aria Fusion.
Software	CFlow vers 1.0.227.4, BD FACSDiva vers 8.0 or 9.0 were used for data collection and FlowJo vers 10.9 was used for data analysis.

Cell population abundance	The purity of tdTomato positive and negative fraction were 96 and 100% respectively and was determined by flow cytometry. Post-sort fractions from MACS bead purification were not re-analyzed.
Gating strategy	Debris was gated out in the SSC-A/FSC-A plot and single cells were gated in the FSC-H/FSC-A plot.<br>Neutrophils in blood were gated as CD11b+Ly6G+, neutrophils in bone marrow as CD45+ CD11b+ Ly6G+ and neutrophils in spleen as CD45+ SSC-hi Gr1-hi. Monocytes in blood were gated as CD11b+ Ly6G- and B and T lymphocytes in blood were gated as CD11b- Ly6G- and CD19+ CD13- or CD19- CD3+ respectively. Common myeloid progenitos were gated as lineage- c-kit+ Sca-1-. Neutrophils, monocytes and macrophages in heart (CD45 MACS bead sorted positive fraction) were gated as SSC-hi Gr1-hi, CD11b-low F4/80- and CD11b-low F4/80+ respectively.<br>FlowJo vers 10.9 was used for data analysis, logical axes were used in all fluorescence plots and Fluorochrome-minus-one (FMO) was used as negative control.

☒ Tick this box to confirm that a figure exemplifying the gating strategy is provided in the Supplementary Information.
